# AMOchar: an amorphous MnOx functionalized biochar to stabilize metal(loid)s in soil and optimize phytostabilization

**DOI:** 10.1038/s41598-025-25164-4

**Published:** 2025-11-21

**Authors:** Sayyeda Hira Hassan, Zubda Zahid, Petr Ouředníček, Jiřina Száková, Dalila Trupiano, Gabriella Stefania Scippa, Lukáš Trakal, Zuzana Vaňková, Manhattan Lebrun

**Affiliations:** 1 Department of Environmental Geosciences, Faculty of Environmental Sciences, Czech University of Life Sciences Prague, Prague 6, Czech Republic; 2 Department of Biosciences and Territory, University of Molise, Pesche, IS Italy; 3 Department of Agroenvironmental Chemistry and Plant Nutrition, Faculty of Agrobiology, Food and Natural Resources, Czech University of Life Sciences Prague, Prague 6, Czech Republic; 4 Université Marie et Louis Pasteur, CNRS, Chrono-Environnement (UMR 6249), 25200 Montbéliard, France

**Keywords:** Biochar impregnation, Metal(loid) immobilization, Soil remediation, Mn leaching, Chemistry, Environmental sciences, Materials science

## Abstract

Biochar (BC) is widely used to immobilize metal(loid)s but its efficiency is not ubiquitous and can be improved via various surface modifications, such as impregnating amorphous Mn oxides (called AMOchars), which showed benefits for metal(loid) immobilization, but also induced considerable Mn leaching. To reduce those drawbacks, we tested two AMOchars, synthesized using molasses (BCM) or sucrose (BCS) as reducing agents, to evaluate the effectiveness of Mn oxide fixation on biochar to reduce metal(loid) transfer to plants and select the most appropriate reducing agent. Soil solution monitoring showed that all materials increased pH and immobilized Cd, Pb, and Zn. The AMOchars further improved Cd and Zn immobilization efficiency while releasing dissolved organic carbon, As, Cu, and Mn. In plants, metal(loid) shoot transfer was reduced by BC (As, Cd, Cu, Mn, and Zn) and BCS (Cd, Pb, and As). To conclude, we demonstrated the effectiveness of Mn oxide fixation on biochar using sucrose to improve phytostabilization. Sucrose as a reducing agent led to a more stable (less organic carbon leaching) material and thus less toxic to the plant. We believe that our study will help raise up the knowledge on biochar modification and make aided phytostabilization process more practiced on the field.

**Supplementary Information:**

The online version contains supplementary material available at 10.1038/s41598-025-25164-4.

## Introduction

Biochar is widely studied for its potential to improve soil properties and immobilize metallic contaminants. Biochar is an alkaline carbon-rich material with a high porosity and a significant specific surface area, it can reduce soil acidity, ameliorate water retention, and sorb metallic contaminants^1^. However, biochar efficiency to sorb metal(loid)s is not ubiquitous; for instance, due to the presence of mainly negatively charged functional groups on its surface, it is generally ineffective towards As^2^. Such dichotomy between cationic and anionic species is an issue when soils are contaminated by both, which is quite common. Moreover, the sorption of cations can be low^3^. To increase the sorption of both cationic and anionic contaminants, the surface of the biochar can be modified through the fixation of additional “functional” groups. Many modifications are possible^4^, providing increased functionality for different chemicals. Studies showed the benefits of Fe functionalization for As^5,6^, while NH_3_ and HNO_3_ functionalization on coconut biochar increased its sorption towards Pb^7^. However, those modifications are often mono-element targeted or only tested in batches^5,8^. When facing a multi-contamination, the difficult part is to find the modification that will be beneficial for all the elements in the soil.

Poorly crystalline Mn oxides (MnOx) or amorphous Mn oxides (AMOs) are the natural inherent part of the soil environment and one of the most important scavengers of metallic contaminants in soils^9^. Due to their amphoteric nature, they are able to strongly bind both cationic and anionic species and were thus successfully tested for the immobilization of metals (Cd, Pb, and Zn) and As in contaminated soils^11–14^. Yet, despite the high sorption efficiency, the oxidative nature of MnOx or AMOs, resulting in an enormous Mn leaching and strong dissolution of soil organic matter (SOM)^13–15^, present the disadvantages of their use. For this reason, our team attempted to prepare the functionalized composites of biochar and amorphous MnOx (called AMOchars) in order to improve the sorption properties of pristine biochar and limit the unwanted effects of MnOx application, such as excessive Mn leaching or SOM dissolution. In this aim, various ways of composite synthesis were tested in order to reach the highest metal(loid) sorption efficiency and lower Mn leaching^16^, while further research was aimed at improving the final synthesis yield and economic costs by the involvement of low-cost sugars (white sugar and waste molasses) in the synthesis process^17^. Although this study provided promising results in batch adsorption tests in terms of the Pb, As, Cd, and Zn sorption capacities, the new materials need to be tested in a complex plant-soil system to evaluate their remediation potential, behavior, and influence on living plants and soil microorganisms. Although some recent studies draw attention to the potential of Mn-functionalized biochars to immobilize/degrade inorganic and organic contaminants^18–21^, they are usually limited to one (or at most two) contaminant(s) and focus mainly on the amendment’s performance toward the targeted pollutant(s), while the information on their interaction with plants is still rather scarce. Furthermore, they often do not consider broader effects on soil and plant properties, particularly the side effects associated with using Mn-based materials, such as potential leaching of Mn or dissolved organic carbon (DOC).

However, the rehabilitation of soils contaminated by metallic elements generally requires the establishment of a vegetation cover, which will reduce soil erosion and leaching, and thus, contaminants transfer to the surrounding environment. In this context, it is crucial to select the proper plant species depending on the intended rehabilitation strategy, *i.e.,* phytoextraction or phytostabilization. In multi-contaminated soils, where complete soil decontamination is difficult to perceive, the phytostabilization approach is often employed to facilitate contaminants’ adsorption on the root surface and/or their precipitation within the rhizosphere and thus inhibit their migration to soil and/or toward the plant aerial part^22^. Considering that toxicants remain in the soil, this strategy is preferentially applicable to heavily contaminated sites and where phytoextraction seems ineffective. On the other hand, it allows the utilization of aerial biomass produced for industrial or agricultural purposes. Several plant species have shown phytostabilization potential. One of them is *Lolium perenne* L., a perennial ryegrass characterized by a rapid growth, an important biomass production, a strong root system, and a tolerance to metallic contaminants^23^. Furthermore, previous studies using this species for phytoremediation purposes have concluded its potential for the revegetation of contaminated soils and the phytostabilization of metallic elements, such as Cu, Cr, and Pb^24–26^ also in the presence of biochar^27^.

Based on these premises, the objective of this study was thus to evaluate the remediation potential of newly synthesized AMOchars in real multi-metal(loid) contaminated soil, taking into account not only the direct immobilization efficiency but also the more complex effects of amendments’ application on (i) the soil physico-chemical characteristics and metal(loid) mobility/ availability, (ii) metal(loid) uptake by perennial ryegrass (*Lolium perenne* L.), (iii) plant performance in term of biomass production, and (iv) soil microbial enzymatic activity. As two types of AMOchar were synthesized using different reducing agents (low-cost sugars), their properties, performance, and stability were compared, including a non-functionalized biochar as a reference.

## Materials and methods

### Amendments synthesis and characterization

For the given study, the two most promising AMOchar composites revealed in our previous study^17^ were synthesized using two different sugar-based reducing agents. The synthesis procedure is described in detail in Supplementary Information (S1). In brief, both composites were prepared using the modified sol–gel synthesis procedure. Here, biochar was added under constant mixing to a 9.3% (w/w) KMnO_4_ solution, and subsequently, 60% (w/w) sucrose or 57% (w/w) molasses solutions were added drop by drop under constant stirring to the reaction mixture, until a black gel was formed. As a result, two types of composites were obtained based on the sugar type used—BCS composite produced using sucrose and BCM composite prepared using molasses. The originated gels were then filtered, air-dried, and ground. The KMnO_4_ and sucrose used were of analytical grade, molasses was provided as a waste product from the production of beet sugar, and its detailed characterization is provided in our previous paper^17^. A commercially available wood biochar with a high specific surface area (Energo Zlatá Olešnice, Czech Republic) was intentionally used in the study to provide a higher active surface for optimal coating with MnOx-phases compared to the straw/grass hay biochar used in the previous study^17^. The prepared materials were subsequently characterized for their physico-chemical properties. The elemental composition was determined using *aqua regia* digestion^28^, followed by the addition of H_2_O_2_ for the decomposition of potential digestion residues. The content of elements in digestate was determined using inductively coupled plasma optical emission spectrometry (ICP-OES; iCAP 7000 series, Thermo Scientific, Germany). The content of total organic (TOC)/inorganic carbon (TIC) was analyzed using the TOC/TC analyzer (TOC-L CPH, Shimadzu, Japan). The pH was measured (ISO 10,390:2021), and the specific surface area (SSA) was determined using the Brunauer–Emmett–Teller (BET) method and the ASAP 2050 instrument (Micrometrics Instrument Corporation, USA).

### Soil sampling and characterization

To test the immobilization potential of newly prepared biochar composites, a multi-metal(loid) contaminated soil from the vicinity of the Příbram non-ferrous metal(loid)s smelter (Czech Republic) was chosen. The particular locality is placed in the alluvium of the Litavka River, north of the village of Trhové Dušníky, about 2.5 km northeast of the Příbram smelter (49.7202056N, 14.0130856E). The alluvium is heavily contaminated with As, Cd, Pb, and Zn, both due to the historical deposition of smelter emissions and also as a consequence of historical occasional seasonal floods bringing the slag material from the slag heaps situated on the riverbank about 3 km upstream^13,29–31^.

The soil samples were collected from the superficial (0 – 20 cm) soil layer across the site to obtain a representative sample, air-dried, sieved through a 2-mm stainless sieve, and basic physicochemical soil characteristics were determined. The soil pH was measured^32^, the particle size distribution was determined using the hydrometric method^33^, and the content of TOC was measured using TOC/TC analyzer (TOC-L CPH, Shimadzu, Japan). The pseudo total elemental composition was determined after *aqua regia* microwave digestion^28^, and the contents of elements in the samples after digestion were measured using ICP-OES. Concerning the fractionation of metals and As, the three-step procedure proposed and validated by the BCR (Community Bureau of Reference) was used to perform sequential extraction aiming at the metallic contaminants^34^ and fractionation of As was determined using As-specific sequential extraction^35^ using the soil collected at the same locality^13^ (Table S1).

### Pot experiment

A pot experiment with perennial ryegrass (*Lolium perenne* L.) grown in contaminated soil amended with biochar and the AMOchar composites was performed to evaluate the potential of newly prepared materials for the immobilization of metal(loid)s in soil and their impacts on soil microbiology and plants themselves. With the soil and the three tested amendments, four experimental variants were prepared: (i) the contaminated soil without amendment (CT), (ii) the contaminated soil amended with the pristine biochar (BC), (iii) the contaminated soil amended with the AMOchar synthesized using sucrose (BCS), and (iv) the contaminated soil amended with the AMOchar synthesized using molasses (BCM). All amendments were added at 2% on a dry-weight basis. The mixture was prepared for each pot, and mixed by shaking to make it homogeneous. For each variant, seven pots containing 250 g of the mixture were prepared and sown with 0.5 g of ryegrass seeds. All pots were placed in the open greenhouse under natural light (the experiment was performed in summer months from July to August) in randomized design and watered regularly (at 70% WHC) with tap drinking water (pH of 7.6, sum of Mg + Ca of 1.04 mmol L^−1^, classified as soft water). The plants were grown for one month, in order to evaluate the benefits and potential toxicity that the AMOchars could have on the first growth stage of the plants, which could greatly affect outcomes in the real field conditions.

#### Soil chemical analysis

When the variants were established, the soil was sampled in each variant to assess the influence of the biochar/biochar composites on metal(loid)s availability. For this, 3 g of soil (with or without amendment) was agitated with 30 mL of 0.01 M CaCl_2_ for 3 h^36^, and the contents of extracted metal(loid)s were determined using ICP-OES.

Furthermore, the chemical properties of soil pore water (SPW) sampled at the beginning (T_0_) and at the end (T_F_) of the experiment were assessed, too. Three hours prior to the SPW collection, the soil was watered with tap water to 70% WHC, and the soil solution was sampled using rhizon samplers (Rhizosphere Research Products, Wageningen, the Netherlands). Collected soil solutions (~ 10 mL) were analyzed directly for pH (pH 7310, WTW, Germany), electrical conductivity (EC), and redox potential (Eh) (Multi 3420, WTW, Germany). The solutions were then diluted to determine the content of dissolved organic carbon (DOC) (TOC-L a, CPH/CPN, Shimadzu) and total nitrogen (TNM-L segment flow analyzer, Shimadzu). Finally, solutions were acidified before elemental analysis with ICP-OES or ICP-MS (ThermoScientific, X series^II^, UK).

At the end of the experiment (TF), also corresponding to the time of ryegrass harvest, part of the soil samples were kept in the fridge for enzymatic analysis (see next Sect. "Soil enzymatic analysis"), and part was air-dried for chemical analysis. Here, the cation exchange capacity (CEC) was measured as the sum of the extractable cations (Ca, Mg, K, Fe, Mn, and Al) in 0.1 M BaCl_2_^37^.

#### Soil enzymatic analysis

The samples kept in the cold were used to measure several enzymes related to element cycles. The enzymes involved in the carbon cycle were β-glucosidase, following the standard protocols^38^, the cellobiohydrolase, measured using methylumbelliferone (MUF) as buffer and p-nitrophenol as substrate, and lipase, using MUF and heptonate^39^. The enzymes of the nitrogen cycle were the alanine aminopeptidase, with MUF buffer and L-alanine-7-amide substrate, the leucine aminopeptidase, using MUF buffer and L-leucine-7-amide substrate, and the chitinase using the depolymerisation method^39^. Finally, one enzyme of the phosphate cycle, the phosphatase, was measured using MUF buffer and p-nitrohenylphosphate as substrate^39^.

#### Plant analysis: chlorophylls content, biomass estimation, and metal(loid)s amount

After one month of growth, ryegrass plants were harvested; three replicates were used for biochemical analysis and four for biomass yield determination and metal(loid) accumulation assessment.

For the biochemical analysis, the aerial biomass was taken fresh, washed with deionized water, and immediately frozen in liquid nitrogen. The samples were kept frozen ( − 20 °C) until they were freeze-dried and crushed. For the determination of the pigment concentrations, 50 mg of crushed samples were vortexed for 1 min with 500 µL ethanol (95%)^40^. After filtration, the absorbance of the supernatant was read at 664 nm and 649 nm. The concentrations of chlorophyll a (Chl a) and chlorophyll b (Chl b) were calculated using Eqs. ^40^ (1) and (2):

1 $${\text{Chl a }}\left( {\mu {\text{g}}.{\text{mL}}^{{ - {1}}} } \right) = {13}.{\text{36 Abs}}_{{{664}}} {-}{5}.{\text{19 Abs}}_{{{649}}}$$

2 $${\text{Chl b }}\left( {\mu {\text{g}}.{\text{mL}}^{{ - {1}}} } \right) = {27}.{\text{43 Abs}}_{{{649}}} {-}{8}.{\text{12 Abs}}_{{{664}}}$$

For the biomass and metal(loid) accumulation determination, the plants were harvested, washed in distilled water, and roots were separated from the aerial parts. Samples were dried for three days at 60 °C to determine dry weight. After, samples were crushed, digested in HNO_3_ + H_2_O_2_, and the contents of metal(loid)s were determined using ICP-OES.

### Statistical analysis

Data were analyzed using the R software. First, the normality and homoscedasticity of the data were assessed using the Shapiro test and the Fligner or Bartlett tests. Means were compared using the ANOVA for parametric data and Kruskal–Wallis for the non-parametric data, followed by a post hoc test. The difference was considered significant at *p* < 0.05.

## Results

### Basic properties of soil and applied amendments

The main physico-chemical properties of contaminated soil collected from the vicinity of the Příbram smelter (Czech Republic) are summarized in Table 1. Briefly, the soil is classified as slightly acidic fluvisol with low CEC and low content of TOC. Concerning the contaminating metal(loid)s, when compared with the limits set by the Ministry of the Agriculture of the Czech Republic (Act No. 153/2016), the limit values are exceeded the most significantly for As (~ 13 times), Cd (~ 70 times), Pb (~ 57 times) and Zn (~ 26 times), with a slightly exceeded limit for Cu.

**Table 1:** Basic physico-chemical characteristics of the studied soils.

pH (H_2_O)	5.95		
pH_KCl_	5.14		
Cation Exchange Capacity (cmol.kg^-1^)	9.1		
Total Organic Carbon content (%)	2.15		
Soil type	Fluvisol		
Particle size distribution (%)			
Clay (%)	7		
Silt (%)	31		
Sand (%)	62		
Texture	Sandy loam		
Pseudo total metal(loid) concentrations (mg.kg^-1^)			Limit values (mg.kg^-1^)
As	267 ± 4		20
Cd	35 ± 0.2		0.5
Cu	87 ± 1		60
Pb	3438 ± 31		60
Zn	3170 ± 31		120
Fe	26 808 ± 241		No limit
Mn	3387 ± 31		No limit

Limit concentrations of metals/metalloids in agricultural soils are set according to the Ministry of the Agriculture of the Czech Republic (Act No. 153/2016).

The fractionation of contaminating metal(loid)s differs significantly based on the type of element (Table S1). A similar fractionation pattern was observed for Cd and Zn when these metals showed the highest relative mobility, with 61% (Cd) and 46% (Zn), respectively, being present in the first most available exchangeable/acid-soluble fraction. These metals are then bound to the soil Fe/Mn oxides (20% for both), some part is present in the residual phase (27% of Zn and 15% of Cd), and just a minor part (3% of Cd and 7% of Zn) is associated to the SOM and sulfides (oxidizable fraction). In contrast, although the pseudo total contents of Pb and Zn are similar, their fractionation is completely different. Just the minimal part of Pb (8%) is present in the first most available fraction, with the prevailing part (61%) bound to soil oxides. Oxidizable fraction is the second most prevailing (20%), and 11% of Pb remains in the residual fraction. Arsenic is also bound relatively strongly in the soil. It is dominantly (87%) associated with Fe and Mn oxides, 72% with amorphous and poorly crystalline ones, and 15% with well-crystalline ones. The 7% of As is then specifically sorbed to other soil phases, and 5% remains in the residual phase.

The basic physico-chemical properties of biochar-based materials (pristine biochar (BC) and AMOchars prepared using molasses (BCM) and sucrose (BCS)) are summarized in Table 2, and more details concerning their elemental composition can be found in Supplementary Information (Table S2). The BC made of waste wood biomass had the highest pH, TOC content and proved significantly the highest specific surface, confirming the high porosity and well-developed structure of the wood-based biochars^41,42^. In both AMOchars (BCM and BCS), the total content of Mn moved around 25%, with a lower active surface caused by functionalization with MnOx layer. Compared to BC, AMOchars also contained lower contents of Al, Ba, Ca, Fe, Mg, P, Ti and Zn, but considerably higher contents of K and S, which remained in the materials as a residue from the synthesis (Table S2).

**Table 2:** Basic physico-chemical properties of applied immobilization amendments.

Amendment	pH	TOC (%)	TIC (%)	Mn (%)	SSA (m^2^ g^-1^)
BC	10.1	71.1 ± 1.0	0.29 ± 0.07	0.044 ± 0.001	442
BCM	9.5	27.4 ± 0.1	0.91 ± 0.01	23.9 ± 0.1	19.6
BCS	8.9	26.6 ± 0.2	0.97 ± 0.00	26.5 ± 0.1	46.9

*TOC* Total organic carbon; *TIC* Total inorganic carbon; *SSA* Specific surface area.

### Soil pore water analysis in pot experiment

Soil pore water (SPW) was collected and analyzed at the start (T_0_) and the end (T_F_) of the experiment to determine the changes in physico-chemical properties and contents of macroelements and metal(loid)s induced by the application of the tested remediation amendments (BC, BCM and BCS) on contaminated soil (CT).

#### Selected physico-chemical properties

At T_0_, SPW pH levels varied significantly among the treatments (Table 3). The application of all biochar materials resulted in a pH increase compared to that of the control. The increase was higher with the AMOchars (1.7 units with BCM and 1.4 units for BCS) than the pristine biochar (0.7 units increase). The pH values remained relatively stable and did not change much until the end of the experiment T_F_.

**Table 3:** Chemical properties of the soil assessed at the beginning (T_0_) and at the end (T_F_) of the experiment in the contaminated soil non amended (CT) or amended with 2% (w/w) of biochar (BC), AMOchar made using molasses (BCM) or AMOchar made using sucrose (BCS).

	pH			Electrical conductivity (mS.cm^−1^)			Redox potential (mV)			Dissolved organic C (mg.L^−1^)			Nitrogen (mg.L^-1^)			CEC (mmol.kg^-1^)
	T_0_	T_F_	Time	T_0_	T_F_	Time	T_0_	T_F_	Time	T_0_	T_F_	Time	T_0_	T_F_	Time	T_F_
CT	6.5 ± 0.4 c	6.6 ± 0.2 c	ns	0.42 ± 0.25 b	6.60 ± 0.18 a	***	443 ± 8 a	551 ± 10 a	***	99 ± 27 b	134 ± 73 b	ns	20 ± 10 b	65 ± 27 a	*	73 ± 4 d
BC	7.2 ± 0.4 b	7.4 ± 0.1 b	ns	0.41 ± 0.35 b	1.56 ± 1.17 c	ns	406 ± 14 b	480 ± 5 b	***	49 ± 16 c	54 ± 11 c	ns	8 ± 2 c	44 ± 23 a	*	82 ± 6 c
BCM	8.2 ± 0.2 a	8.0 ± 0.2 a	ns	1.43 ± 0.22 a	6.96 ± 3.50 ab	*	383 ± 11 c	465 ± 26 c	**	693 ± 374 a	873 ± 759 a	ns	53 ± 17 a	83 ± 56 a	ns	95 ± 2 b
BCS	7.9 ± 0.5 a	8.0 ± 0.3 a	ns	0.96 ± 0.35 a	4.39 ± 2.90 b	ns	394 ± 4 bc	441 ± 21 c	**	396 ± 197 a	890 ± 886 a	ns	39 ± 19 a	61 ± 64 a	ns	123 ± 31 a

pH Electrical conductivity, dissolved organic C content and nitrogen content were measured in soil pore water, while cation exchange capacity (CEC) was evaluated in the soil. Letters indicate significant differences between treatments within each sampling time (*p* < 0.05) (n = 5). Time effect is shown by *p < 0.05, **p < 0.01, ***p < 0.001, and ns = non-significant.

Effect sizes: pH = 0.756 (T0) & 0.817 (TF); Electrical conductivity = 0.647 (T0) & 0.502 (TF); Redox potential = 0.740 (T0) & 0.754 (TF); Dissolved organic C = 0.811 (T0) & 0.659 (TF); Nitrogen = 0.767 (T0) & − 0.132 (TF); CEC = 0.888 (TF). Confidence intervals: pH = 0.627–0.904 (T0) & 0.658–0.918 (TF) ; Electrical conductivity = 0.466–0.862 (T0) & 0.339–0.836 (TF) ; Redox potential = 0.511–0.94 (T0) & 0.534–0.912 (TF) ; Dissolved organic C = 0.669–0.911 (T0) & 0.443–0.897 (TF) ; Nitrogen = 0.575–0.919 (T0) & 0.0166–0.547 (TF) ; CEC = 0.777–0.943 (TF).

At T_0_, the electrical conductivity (EC) of the SPW was significantly higher with the AMOchars (BCS: 0.96 ± 0.35 mS.cm^−1^, BCM: 1.43 ± 0.22 mS.cm^−1^) compared with the pristine biochar (BC: 0.41 ± 0.35 mS.cm^−1^) and the control (CT: 0.42 ± 0.25 mS.cm^−1^) (Table 3). The EC values generally increased towards the end of the experiment (T_F_), with control values reaching those of AMOchars and the lowest EC value recorded for pristine biochar.

The SPW redox potential (Eh) suggested oxidative conditions in all variants, with an increasing trend in time (T_0_ vs. T_F_) (Table 3). Generally, the highest Eh values were recorded both at the beginning and at the end of the experiment for control (443 and 551 mV, respectively), and the lowest values for AMOchars. This trend is opposite to the recorded pH, showing the common inverse relationship of the pH/Eh values.

#### Macroelements

The application of both AMOchars promoted a significant increase in dissolved organic carbon (DOC) concentrations (Table 3), by sevenfold and fourfold in BCM and BCS, respectively, at the T_0_ compared to the control (99 mg L^−1^), without significant difference between BCS and BCM. On the contrary, the application of pristine biochar reduced OC leaching by 51%. The same trend was observed at the end of the experiment, with a decrease of 60% in the BC treatment and an increase of 6.6- and 6.5-fold in the BCM and BCS treatments, compared to the control, and still no significant difference between the two AMOchars.

Also, the concentrations of total nitrogen (TN) (Table 3) were increased by the presence of AMOchars (yet not so significantly as in the case of DOC) and decreased by the pristine biochar, but this trend was observable only at the beginning of the experiment (T_0_). In the end, there were no significant differences among the treatments.

#### Concentrations of metal(loid)s

**Fig. 1:**
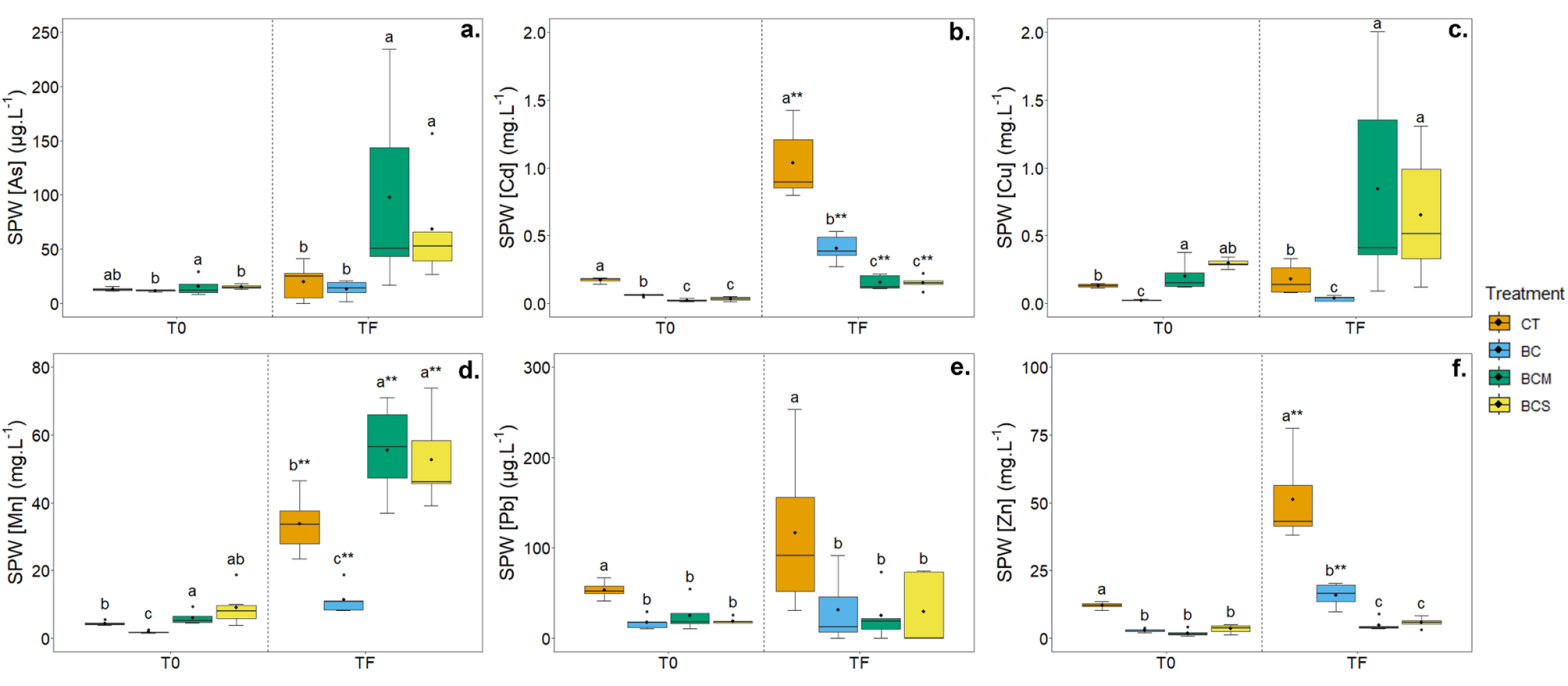
Soil pore water concentrations of As (**a**), Cd (**b**), Cu (**c**), Mn (**d**), Pb (**e**) and Zn (**f**) measured at the beginning (T0) and at the end (TF) of the experiment on the contaminated soil non amended (CT) or amended with 2% (w/w) of biochar (BC), AMOchar made using molasses (BCM) or AMOchar made using sucrose (BCS). Letters indicate significant differences between treatments (*p* < 0.05) (n = 5). Difference within the same treatment between T0 and TF is shown with *p < 0.05, **p < 0.01, and ***p < 0.001, next to the statistical letters of TF. Effect sizes: As = 0.321 (T0) & 0.464 (TF); Cd = 0.827 (T0) & 0.819 (TF); Cu = 0.759 (T0) & 0.659 (TF); Mn = 0.652 (T0) & 0.699 (TF); Pb = 0.463 (T0) & 0.215 (TF); Zn = 0.622 (T0) & 0.826 (TF). Confidence intervals: As = 0.22–0.819 (T0) & 0.321–0.824 (TF) ; Cd = 0.695–0.927 (T0) & 0.663–0.93 (TF) ; Cu = 0.592–0.914 (T0) & 0.445–0.897 (TF) ; Mn = 0.417–0.905 (T0) & 0.494–0.908 (TF) ; Pb = 0.321–0.82 (T0) & 0.125–0.74 (TF) ; Zn = 0.385–0.902 (T0) & 0.668–0.939 (TF).

Concentrations of metal(loid)s in SPW are summarized in Fig. 1. The As concentration (Fig. 1a) increased in control from 13.1 at T_0_ to 19.9 µg.L^−1^ at T_F,_ and an increasing trend with time was also observed for other variants, yet the difference was not statistically significant. When looking at the effects of different treatments, the amounts of released As were relatively similar at the beginning of the experiment, yet increased significantly for AMOchars towards the end of the experiment, compared to control and pristine biochar.

The effect of amendments application on the release of Cd (Fig. 1b) and Zn (Fig. 1f) had almost the same pattern, just with the difference in the scale of concentrations (range of µg.L^−1^ for Cd and tens of mg.L^−1^ for Zn) (Fig. 1), with Zn being the most abundant contaminating element in the SPW. For both metals and times of SPW collection, concentrations of Cd and Zn were significantly highest in control treatments and were efficiently decreased by the application of all biochar-based materials, with the particular effect of AMOchars, which sustained over the whole time of the experiment. At T_F_, pristine biochar was able to decrease the metal concentrations compared to control by 61% and 69% for Cd and Zn, while BCS by 85% and 90%, and BCM by 85% and 89%, respectively.

In contrast, Cu mobility (Fig. 1c) at T_0_ was significantly decreased (by 5.6-fold) when pristine biochar was added. In contrast, the BCM increased the SPW Cu concentration by more than two-fold, while no significant effect was observed in BCS (Fig. 1). By T_F_, BC reduced Cu concentration by 80% in SPW, while the addition of both AMOchars mobilized Cu even more significantly than at the beginning of the experiment (4.7 and 3.6-fold increase with BCM and BCS, respectively).

Concerning Pb (Fig. 1e), despite its high pseudo total content in soil (Table 1), its concentration in SPW was relatively low, mainly in the order of tens of µg.L^−1^. The highest Pb concentrations in SPW were reached in the contaminated soil without amendment (53 ± 9 µg.L^−1^ at T_0_ and 117 ± 89 µg.L^−1^ at T_F_). At both the beginning and the end of the experiment, all the biochar materials (BC, BCM and BCS) immobilized Pb to a similar extent, reaching a ~ fourfold decrease in SPW Pb concentrations at T_F_.

As the applied AMOchars contain a significant portion of Mn (Fig. 1d), its concentrations in SPW were monitored, too. Similarly, as in the case of Cd and Zn, the amount of released Mn significantly increased at the end of the experiment compared to the beginning, even in the control soil. At T_0_, the application of pristine biochar reduced SPW Mn concentration by 58% compared to control (4.4 mg.L^−1^), while the AMOchars increased it by 1.4-fold (BCM) and 2.0-fold (BCS). By T_F_, Mn concentrations increased significantly in all treatments compared to the beginning of the experiment. A similar trend was observed at the end of the experiment, with no statistical difference between the types of AMOchar used (BCM or BCS).

#### Metal(loid)s availability and CEC

**Fig. 2:**
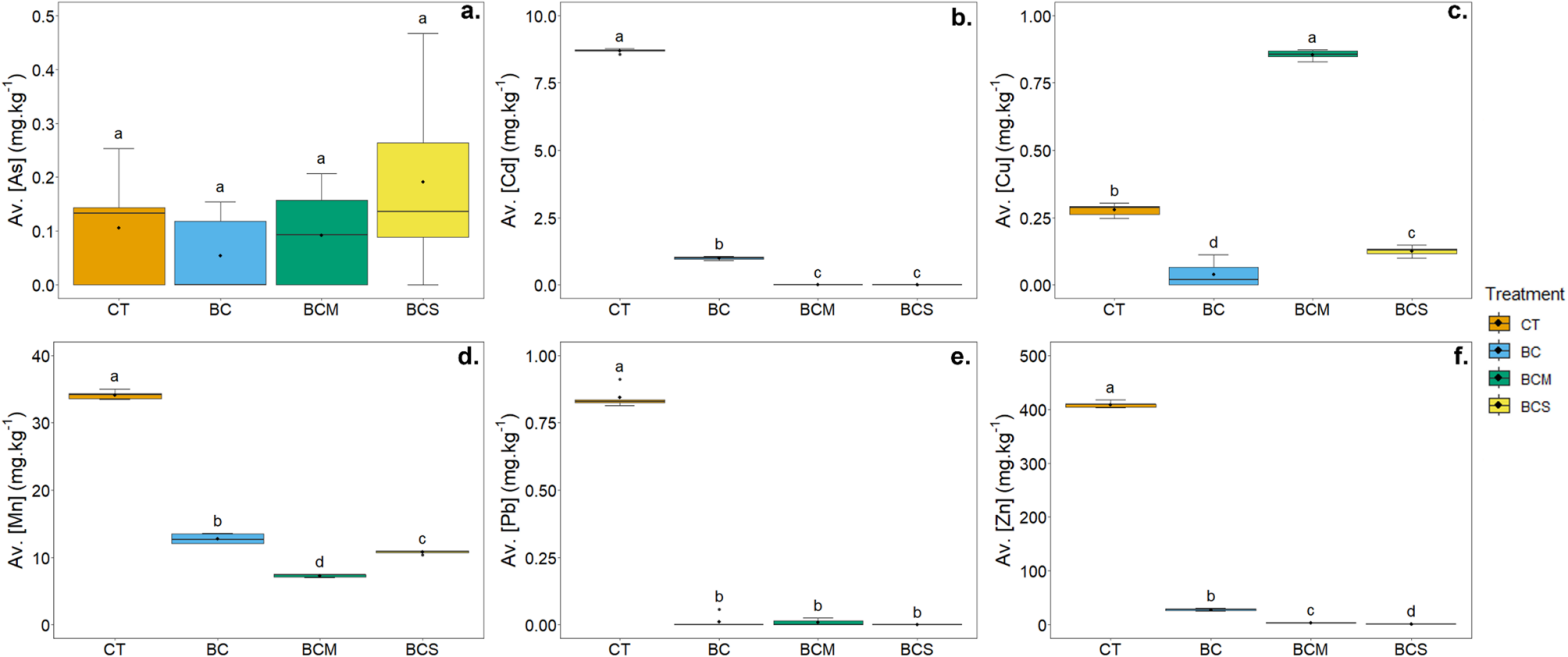
Available concentrations of As (**a**), Cd (**b**), Cu (**c**), Mn (**d**), Pb (**e**), and Zn (**f**) measured at the beginning of the experiment in the contaminated soil non amended (CT) or amended with 2% (w/w) of biochar (BC), AMOchar made using molasses (BCM) or AMOchar made using sucrose (BCS). Letters indicate significant differences between treatments (*p* < 0.05) (n = 5). Effect sizes: As = − 0.0585; Cd = 0.870; Cu = 0.912; Mn = 0.929; Pb = 0.679; Zn = 0.929. Confidence intervals: As = 0.0273–0.591; Cd = 0.765–0.949 ; Cu = 0.817–0.946 ; Mn = 0.856–0.947; Pb = 0.439–0.917 ; Zn = 0.863–0.947.

Besides the direct soil solution sampling, the availability/exchangeable fraction of present contaminants at the beginning of the experiment was evaluated using the 0.01 M CaCl₂ extraction (Fig. 2).

In some cases (*e.g.,* Cd, Pb, Zn), the changes in metal(loid) availability showed similar trends as the concentrations in soil solution (Fig. 1), yet with a much stronger stabilization effect, decreasing the concentrations of Cd (Fig. 2b), Pb (Fig. 2e), and Zn (Fig. 2f) in extracts from AMOchars-amended soils even below the limit of detection. In contrast, Cu (Fig. 2c), and especially Mn (Fig. 2d), showed different extractability patterns than what was observed in the SPW. In the case of Cu, BCM supported the Cu availability (a threefold increase compared to control), yet the application of BCS decreased the extracted Cu amount by 55%. For Mn, interestingly, the lowest extracted amounts were reached with both the AMOchars, showing even lower concentrations than pristine biochar.

In addition, the influence of applied amendments on soil CEC was investigated at the end of the experiment (Table 3). The CEC values varied significantly among the treatments. The lowest value was measured in contaminated soil (73 ± 4 mmol kg^−1^), while it increased in biochar-amended soil: BCS (68%) > BCM (30%) > BC (12%).

### Substrate enzymatic activity

The activity of different enzymes related to C, N, and P cycles was analyzed in all soil substrates (CT, BC, BCM and BCS) at the end (TF) of the experiment (Table 4). All the enzymes related to C cycle, *i.e*., β-glucosidase, lipase, and cellobiohydrolase, and P cycle, phosphatase, were not affected by the application of biochars, either pristine or modified. However, we can note that phosphatase activity was higher in BCM than BCS variant. Conversely, two out of the three enzymes related to the N cycle were affected. In detail, chitinase activity was not changed, while alanine aminopeptidase was increased by the pristine biochar amendment, and leucine aminopeptidase was increased by both the pristine biochar and the BCM AMOchar.

**Table 4:** Enzyme activities (nanomole.min^-1^.g^-1^) measured at the end of the experiment in the contaminated soil non amended (CT) or amended with 2% (w/w) of biochar (BC), AMOchar made using molasses (BCM) or AMOchar made using sucrose (BCS).

	β-glucosidase	Cellobiohydrolase	Lipase	Alanine aminopeptidase	Leucine aminopeptidase	Chitinase	Phosphatase
CT	0.63 ± 0.54 a	0.08 ± 0.07 a	17.41 ± 8.56 a	0.76 ± 0.46 b	0.55 ± 0.31 b	0.20 ± 0.08 a	0.74 ± 0.66 ab
BC	0.56 ± 0.25 a	0.19 ± 0.24 a	17.87 ± 4.99 a	1.53 ± 0.63 a	1.28 ± 0.51 a	0.17 ± 0.05 a	0.36 ± 0.18 ab
BCM	0.50 ± 0.33 a	0.07 ± 0.05 a	15.29 ± 5.65 a	1.21 ± 0.29 ab	1.03 ± 0.31 a	0.23 ± 0.09 a	0.87 ± 0.23 a
BCS	0.42 ± 0.25 a	0.07 ± 0.04 a	14.77 ± 2.93 a	0.97 ± 0.25 ab	0.86 ± 0.23 ab	0.19 ± 0.09 a	0.24 ± 0.35 b

Letters indicate significant differences between treatments (*p* < 0.05) (n = 5).

Effect sizes: β-glucosidase = − 0.139; Cellobiohydrolase = − 0.0980; Lipase = − 0.106; Alanine aminopeptidase = 0.187; Leucine aminopeptidase = 0.274; Chitinase = − 0.115; Phosphatase = 0.174. Confidence intervals: β-glucosidase = 0.0192–0.599 ; Cellobiohydrolase = 0.025–0.605 ; Lipase = 0.0223–0.627 ; Alanine aminopeptidase = 0.0693–0.866 ; Leucine aminopeptidase = 0.0999–0.835 ; Chitinase = 0.018–0.61; Phosphatase = 0.0883–0.737.

### Plant yield, pigments, and metal(loid) uptake on tested growing substrates

**Fig. 3:**
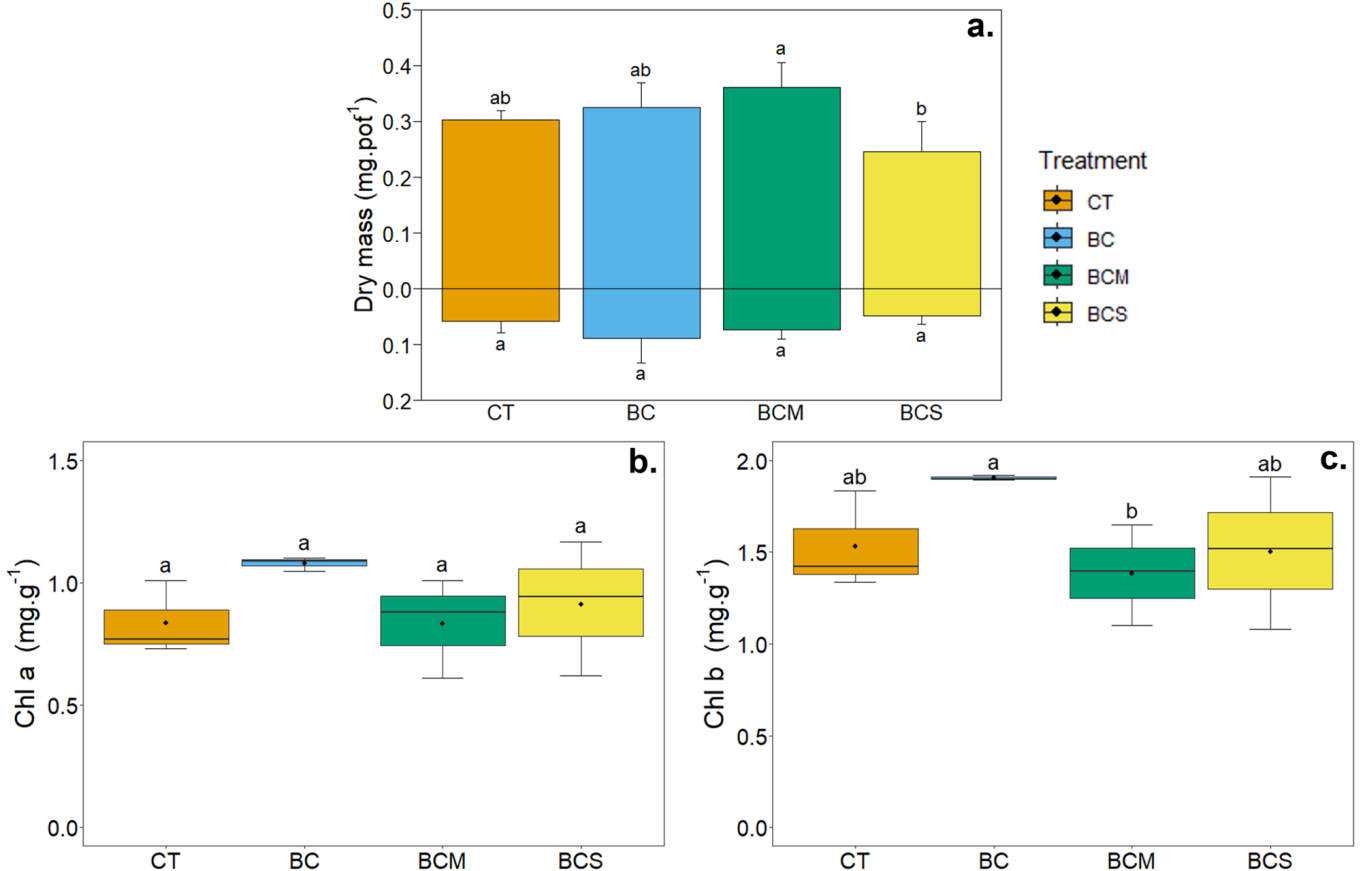
Biomass production of the shoot and roots (**a**) and shoot chlorophyll contents (**b**, **c**) of *Lolium perenne* after 1 month of growth in the contaminated soil non amended (CT) or amended with 2% (w/w) of biochar (BC), AMOchar made using molasses (BCM) or AMOchar made using sucrose (BCS). Letters indicate significant differences between treatments (*p* < 0.05) (n = 3–4). Effect sizes: Shoot dry mass = 0.353; Root dry mass = 0.181; Chl a = 0.173; Chl b = 0.23. Confidence intervals: Shoot dry mass = 0.163–0.902; Root dry mass = 0.0937–0.886; Ch a = 0.159–0.93; Chl b = 0.166–0.913.

In the control soil, plants produced 302 mg.pot^−1^ of dry aerial biomass and 58 mg.pot^−1^ of dry root biomass (Fig. 3a). The highest aerial biomass yield was obtained from the BCM treatment (361 mg.pot^−1^), while the lowest was with the BCS treatment (246 mg.pot^−1^), but none of the treatments differed significantly from the control. Also, the differences in the root biomass were not significant, with the highest mass recorded for BC (89 mg.pot^−1^) and the lowest for BCS (49 mg.pot^−1^). The concentrations of pigments (chlorophyll a and b) in plant leaves under different treatments are presented in Fig. 3b and c. The highest contents for both chlorophylls were recorded in the treatment with BC, yet the difference was statistically significant only in the case of chlorophyll a. Generally, pigment concentrations were relatively similar among the control and AMOchar treatments, with slightly higher chlorophyll a and b contents for BCS than BCM.

**Fig. 4:**
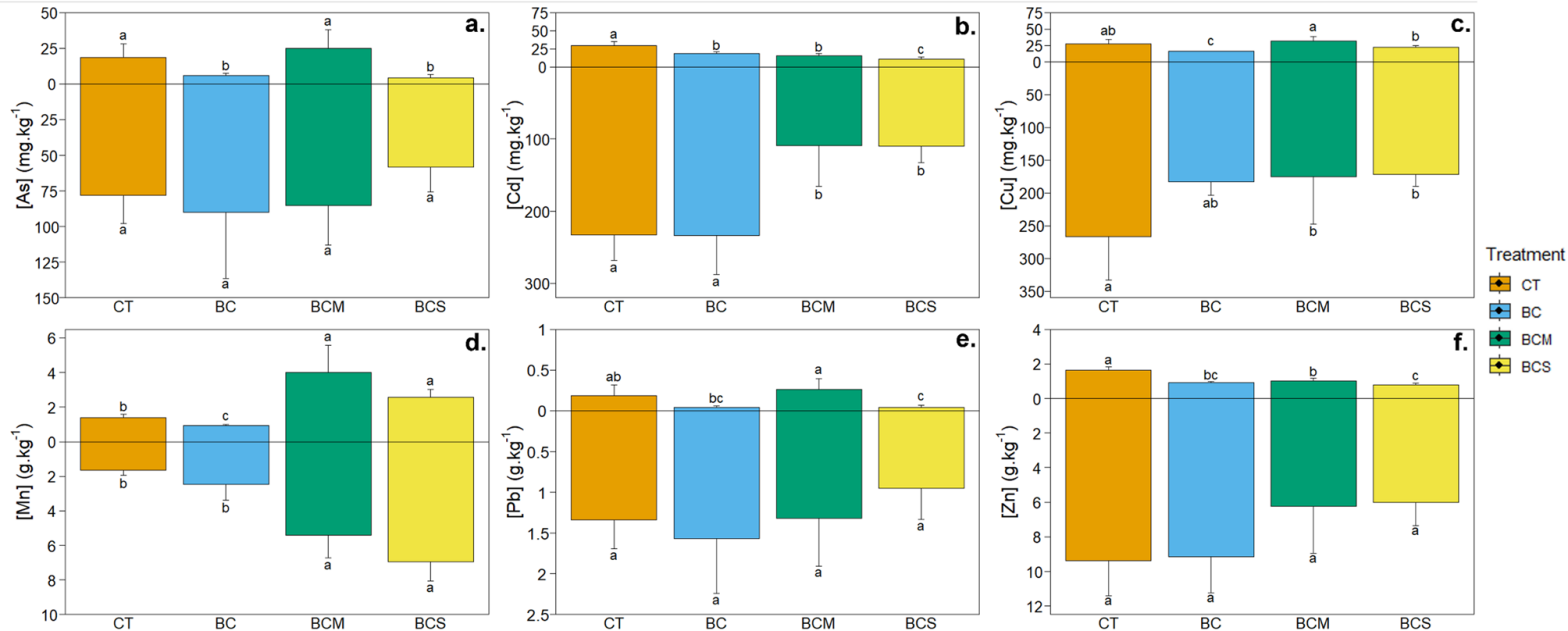
Concentrations of As (**a**), Cd (**b**), Cu (**c**), Mn (**d**), Pb (**e**), and Zn (**f**) in the shoot and roots of *Lolium perenne* after 1 month of growth in the contaminated soil non amended (CT) or amended with 2% (w/w) of biochar (BC), AMOchar made using molasses (BCM) or AMOchar made using sucrose (BCS). Letters indicate significant differences between treatments (p < 0.05) (n = 4). Effect sizes: As = 0.713 (Shoot) & -0.0383 (Root); Cd = 0.732 (Shoot) & 0.644 (Root); Cu = 0.695 (Shoot) & 0.270 (Root); Mn = 0.834 (Shoot) & 0.805 (Root); Pb = 0.522 (Shoot) & 0.0583 (Root); Zn = 0.607 (Shoot) & 0.217 (Root). Confidence intervals: As = 0.6–0.909 (Shoot) & 0.0459–0.821 (Root) ; Cd = 0.528–0.937 (Shoot) & 0.49–0.923 (Root) ; Cu = 0.428–0.927 (Shoot) & 0.136–0.903 (Root) ; Mn = 0.625–0.949 (Shoot) & 0.614–0.945 (Root) ; Pb = 0.351–0.88 (Shoot) & 0.0934–0.822 (Root) ; Zn = 0.344–0.92 (Shoot) & 0.114–0.867 (Root).

The contents of metal(loid)s in roots and shoots of ryegrass are depicted in Fig. 4. Generally, significantly higher metallic contents were present in roots than in shoots, with the only exception of Mn (Fig. 4d) in control, where the Mn concentration in shoots (1800 mg kg^−1^) was higher than in roots (1660 mg kg^−1^). The proportion of Mn transported to aboveground biomass was even in other treatments relatively higher than the translocated amounts of other (metal)loids.

With respect to the effect of applied amendments on contaminants uptake, generally, the most effective treatment was the BCS, which was able to decrease the contents of As, Cd, Cu, Pb, and Zn in shoots by 73%, 66%, 34%, 76%, and 54%, respectively. Interestingly, although its effects on the availability of these metal(loid)s in soil and SPW were usually similar to the second type of AMOchar, the BCM was not able to reduce the concentrations of As and Pb below the control level, which is in contrast to a significant effect of BCS. Besides the BCS, also BC was highly effective, reducing the contents of As, Cd, Cu, Pb, and Zn in aboveground biomass by 65%, 39%, 43%, 74%, and 45%, respectively. The BCS was also the most effective amendment in decreasing the contents of targeted metal(loid)s in plant roots, where its advantage over pristine biochar was even more significant, reducing the root As, Cd, Cu, Pb, and Zn contents by 35%, 53%, 7%, 39%, and 33% compared to simple BC.

Finally, the influence on Mn dynamics in plants was investigated as well (Fig. 4d). Application of both AMOchars led to the increased uptake of Mn, resulting in a 1.7-fold (BCS) and 2.2-fold (BCM) higher content of Mn in shoots and 4.2-fold (BCS) and 3.3-fold (BCM) contents in roots compared to control.

## Discussion

### Soil health (pH, DOC, nutrients) and enzyme activities

The application of the biochar materials increased the pH of the soil, as has been observed in previous studies^43–45^. Biochar amendments have been demonstrated to increase soil pH, especially when the soil has an acidic pH^46^, which was our case. Two main properties of the biochar can explain a decrease in soil acidity. First, biochar has an alkaline pH (between 8.9 and 10.1 in our case), which will buffer soil pH, especially in coarser texture soils^47^. Second, the surface of the biochars should contain functional groups, mainly negatively charged, that can bind H^+^^43^. When the modified biochars (AMOchars) were applied, soil pH further increased, which contradicts the study of Tan et al.^48^, where the application of biochar and Fe–Mn biochar led to a similar increase in soil pH. In our case, the higher pH increase in soils amended with AMOchars than pristine biochar can be explained as a combined effect of proton adsorption on the AMOchar surface and additional proton consumption caused by the hydrolytic dissolution of the MnOx layer^15^. The higher soil pH promoted by the addition of AMOchars is also a key parameter for the immobilization of contaminating elements in the soil.

All biochar materials also showed to be effective in increasing soil CEC, demonstrating a higher capacity to bind cations, including nutrients, and thus an improvement of soil fertility. Moreover, a higher CEC will allow for a stabilization of pollutant cations such as Cd and Pb. Soil CEC is linked to soil pH, thus the increase could be related to the improvement of soil pH^49^. In addition, organic matter was shown to bind cations and thus to contribute to the soil CEC^49^. As the CEC was higher with the AMOchars than the biochar, together with the soil pH and DOC, those two mechanisms seem to have contributed. Finally, the higher CEC with the AMOchar BCS compared to BCM could be related to their specific surface area, which is higher in the case of BCS, leading to potentially more adsorption sites for cations, and thus a higher CEC^50^.

When looking at macroelements, a dichotomy was observed between the pristine biochar and the AMOchars. While the biochar lowered their leaching from the soil, the AMOchars induced an important release of organic C and N. Biochar is made of recalcitrant carbon^51^. Therefore, the carbon added to the soil through biochar application is usually stabilized in the soil and does not leach. Moreover, biochar has a high sorption capacity. Studies have shown its capacity to sorb N (in nitrate and/or ammonium form)^52^ and stabilize it in the soil^53^. In addition to direct sorption, biochar could stabilize C and N through the improvement of the physical properties of the soil, *i.e*., bulk density, porosity, and structure and stability of aggregates. It has been shown that increasing the stability of the aggregates in the soil improved the stability of organic matter in the soil as well^54^. Although it was not measured here, studies have shown the positive effects of biochar on such physical soil properties^55,56^. Contrary to biochar, the AMOchars led to an important release of DOC into the soil solution, as was observed in our previous study^15^. One of the reasons for such intense OC leaching is the chemical composition of the AMOchars. After the reduction and fixation of Mn oxides on the biochar surface, even though the materials are heavily rinsed, some sugar residues can persist and be released once incorporated into the soil. In addition, Mn oxides’ presence on the biochar surface causes the oxidation of the soil organic matter and, therefore, its dissolution. Such a release of organic carbon can have a drastic influence on metal(loid) behavior, as DOC can form complexes with metal(loid)s, rendering them more soluble^57^.

Similarly to macroelements, biochar and AMOchars had contrasting effects on the soil enzyme activities. Although none of them affected C and P-related enzymes, the pristine biochar tended to increase the activities of the enzymes of the N cycle, while the AMOchars had no effect compared to the control soil. Due to the rise of DOC and pH induced by the biochar and AMOchars, we expected to have an increase in soil enzyme activities, especially of the C cycle, as the labile carbon of biochar could be used as an energy source by microorganisms^58,59^. Moreover, in general, when carbon-rich materials are added to the soil, N-related enzymes are promoted^60,61^, to maintain the C/N balance of the microorganisms. However, as the carbon added by biochar is, for the most part, recalcitrant and thus not accessible for microorganisms, such a mechanism seems to be unlikely, which is confirmed by the none effect on C cycle enzymes. Another possible explanation is that the biochar pores serve as a habitat for the microorganisms^62^, which then find themselves in contact with the nitrogen fixed on the biochar, and thus can consume it. Finally, biochar, through its influence on soil properties and the changes it induced, may have altered the microbial community structure^58,59^, thereby affecting its functions. Compared to biochar, the application of AMOchars did not induce significant changes in enzyme activities, even though it added high amounts of easily degradable C and N. We can thus hypothesize that the application of these materials did not induce a drastic change in the microbial community or in root exudation to the point of affecting its functioning, in contrast to biochar.

### Immobilization of metal(loid)s in soil

Up to date, there exist relatively few studies investigating the immobilization effects of Mn-functionalized biochars directly in soil, focusing mainly on single elements, such as As^63,64^ or Cd^48,65^ or two at maximum (*e.g*., Pb and Hg)^21^. In our case, the soil originating from the historical mining and smelting locality is heavily contaminated with metals (mainly Cd, Pb and Zn and, to a smaller extent, Cu) and also with metalloid As. Besides the high pseudo total contents of given elements, a significant portion of especially Cd (24 mg.kg^−1^) and Zn (1822 mg.kg^−1^) is present in the easily mobilizable fraction as shown by the results of sequential extraction (Table S1). Based on these results and other studies investigating the same locality^14,66^, Zn is the main contaminant dominating the soil solution and limiting plant growth. Considering, in addition, the slightly acidic pH that further promotes the solubility of metallic contaminants, remediation of such soil presents a challenge.

When looking at the elemental concentrations in SPW (Fig. 1), it is visible that no amendment, nor pristine biochar or AMOchars, presents the most efficient option for all of the targeted elements at the same time. Instead, the overall stabilization efficiency towards particular metal(loid)s differs in relation to the involved immobilization mechanisms, depending on the type of element and its fractionation and binding in soil. As mentioned above, Cd and Zn can be considered the most concerning ones in terms of their solubility in the studied soil. These two elements have generally similar geochemical behaviour^67^, and showed in our previous studies similar leaching patterns when various stabilizing agents were added to the soil^13,15^. From various soil physico-chemical parameters, pH was shown to be the main factor driving the mobility of these elements not just in this particular soil^15^, but also in soils studied by different authors^68,69^, which corresponds to their prevailing presence in the exchangeable + acid-soluble fraction. This statement is in accordance with the observations obtained in the current study, when the highest pH increase after the AMOchar’s addition was followed by the highest decrease of Cd or Zn concentrations in the SPW. Concerning Pb, despite its high total load in the soil (∼3400 mg.kg^−1^), which was similar to that of the Zn (∼3200 mg.kg^−1^), its concentration in the SPW was about two orders of magnitude lower. Also, the Pb SPW concentrations (Fig. 1e) are related to the changes in pH^15^, yet in contrast to the Cd and Zn, the same efficiency was observed for pristine biochar and AMOchars, despite the pH differences between the materials and the fact, that MnOx functionalization usually rather increases the material’s sorption capacity for Pb compared to the pristine biochar^16,21,70^. A possible explanation could be a counterpart remobilization process, when part of Pb originally bound to SOM (705 ± 93 mg.kg^−1^ present in oxidizable fraction; Table S1) is released due to SOM oxidation and dissolution after the AMOchar addition, resulting in a similar final Pb leaching for pure biochar and AMOchars. The results for Cu are interesting, as they demonstrate the specificity of the soil environment compared to simple batch adsorption testing. Although Mn-based functionalization of biochars has shown to be efficient in increasing the sorption capacities for Cu in simple model sorption systems^71,72^, when applied to soil, it acts rather as a Cu leaching promoter^11^. In contrast to Cd, Pb, and Zn, which are mainly pH-driven, Cu’s mobility is related mainly to the SOM dynamics, due to its specific affinity for SOM and the formation of organic complexes^73,74^. For this reason, the highest Cu leaching was recorded in the variants amended with AMOchars, correspondingly to the increased levels of DOC. Studies have shown the affinity of DOC and Cu^75,76^, and especially the fulvic and humic acids present in DOC^76,77^. Although this last part would require deeper molecular analysis to be confirmed. However, the role of DOC in the solubilization of Cu is supported by the higher Cu levels associated with the pH of the soil solution, which remained slightly alkaline throughout the experiment. This condition promoted more deprotonated functional groups on the DOC surface, thereby increasing the affinity of Cu for DOC^75,76^ and ultimately resulting in a greater release of Cu over time (*i.e*., a higher Cu concentration at the end of the experiment). In the case of As, the results from SPW were also not consistent with the data from adsorption experiments, where pristine biochar showed virtually no binding capacity for As (V) in contrast to highly efficient MnOx-biochar composites^16,17^. Yet, when applied to soil, the addition of AMOchars rather promoted As release, similarly as in the case of Cu, while pristine biochar showed no effect compared to control. The increased As leaching could be related both to the pH increase after the AMOchars addition supporting the As solubility, and also to the increased leaching of DOC, which was found as a supporting factor for As release and transport^78,79^. The behavior of Mn depended on the material. Biochar was able to immobilize Mn and thus reduce its SPW concentration, thanks to its sorption capacity and the rise in soil pH^80^. On the contrary, following the AMOchar application, SPW Mn was highly increased, showing that part of the Mn fixed on the biochar surface was released. Such release of Mn was observed in our previous study^13^, but to a much higher extent. The release of Mn could have significant ecotoxicological impacts^57,81^: (i) chemical properties can be altered, impacting vegetation and crops; (ii) it has adverse effects on aquatic invertebrates, disrupting their immune system, and (iii) on humans, its main targets are the central nervous system, the reproductive system and the immune system, where it causes function disruption. Consequently, solutions need to be find to prevent such damages. One of them is to optimize the AMOchar synthesis process to render Mn more stable. Another possibility is to mix AMOchar with a pristine biochar. Finally, another solution is to combine AMOchar with a Mn accumulating plant, such as *Grevillea meisneri*.

### Plant growth and metal(loid) uptake

The benefits of biochar materials for soil fertility, and consequently for plant growth and physiological health, have been demonstrated multiple times in the context of metallic contamination^45,82^. Such an amelioration of the plant condition is generally related to an immobilization of the contaminants, and thus a reduction of their toxicity, an improvement of the soil physical structure (*e.g*., reduced bulk density, increased aggregate stability), and a rise in nutrient content and availability. Although our soil data demonstrates an immobilization of several of the main metallic elements, our materials were not able to improve plant biomass production and chlorophyll content, even after the fixation of Mn oxides on the biochar surface. Even if biochar generally ameliorates plant development, some studies also showed no significant effects^83,84^. It is possible that, although the soil presented a high level of metallic contamination, its physicochemical properties were sufficient to allow ryegrass to grow. Moreover, we can note that although the AMOchars induced an important leaching of As and, more importantly, Mn, this did not negatively affect plant development, as there was no reduction in biomass production or chlorophyll content, indicating no impairment of the photosynthetic machinery.

Regarding the uptake of the targeted contaminants, it did not every time correspond to their concentrations in SPW. While Cd and Zn showed a similar uptake pattern as the SPW with the most efficient treatment of BCS, the Pb revealed a high efficiency of BCS but a high transfer to plants aerial tissues with BCM, even though this composite reduced SPW concentrations. Such results contradict the work of Álvarez-Rogel et al.^85^, demonstrating a correlation between the reduction in Pb contents in soil pore water and its translocation to the plants. On the contrary, the study of Wen et al.^86^ showed similar trends as observed in this study, with a decrease in the availability of Pb with Fe-modified biochar but an increase in Pb translocation to rice plants. Similarly, Rees et al.^87^ observed an increase in metal (Cd and Zn) accumulation in the hyperaccumulator *Nocceae caerulescens*, but also in *Lolium perenne* after the application of biochar, even though that material reduced metal concentration in the soil solution. We could explain such a higher accumulation by the potential release of the previously sorbed Pb from AMOchar surfaces, as it has been discussed for the soil solution concentrations. However, as both AMOchars presented similar SPW concentrations but only BCM failed to reduce Pb translocation to plants, that mechanism seems unlikely. Similarly, the reason given by Rees et al.^87^ for a reduction of competition between the immobilized major cations and Pb seems to not have been the major contributor, as both AMOchars immobilized cations. However, their hypothesis regarding a modification of the cell wall composition requires a deeper analysis to understand why only Pb would pass through this barrier and not the other elements. In addition to this possible mechanism, we can hypothesize that the higher Pb accumulation with BCM was related to a modification of the Pb speciation, and/or a modification of the plant internal machinery. Element uptake is not only dependent on the element itself (some are transported via passive transport, other by active transport) and its content in the soil but also on the form in which the element is found in the soil. For instance, the elements in free ionic forms are more easily assimilable by the plants than those complexed to humic or fulvic acids^88^. It has also been shown that the shoot metal(loid) content increased with the concentration of dissolved organic matter in the soil^89^, as we observed. In the case of Pb, it is mainly transported from the soil to the aerial part through a passive pathway (even though it can use some non-specific transporters, such as Ca^2+^ channels^90^), and in an ionic form (Pb^2+^)^91^. It is possible that although BCM amendment reduced SPW Pb concentration, this Pb was found in a specific form (*e.g*., DOC-Pb complex) that could be taken up by the plants. A more detailed study about the speciation of the elements in the soil will need to be performed to confirm this hypothesis. In addition, the plants possess pathways borrowed by the metallic elements to enter the plant and translocate into the aerial part. When looking at the other elements, we can even see that although root concentrations are similar between all biochar treatments, their translocation tends to be higher with the BCM AMOchar. This testifies to a modification of the translocation pathway within the plant in response to that specific amendment. It is especially the case for As, which has chemical similarities with P, and thus can use its transporters. Therefore, although SPW As concentrations were lower, it could still pass into the plants. More work needs to be done to understand which are those pathways and why they are more affected by BCM than BCS or pristine biochar.

For Cd, Pb, and Zn, the decrease in accumulation can be related primarily to their immobilization in the soil. Yet, the most interesting results were recorded for As and Cu, where BCS increased their concentrations in SPW but simultaneously reduced their transfer to the plant. Such immobilization and transfer behavior matches the results of Beesley et al.^2^, who showed important leaching of As from soil following biochar amendment but a reduction of As transfer to tomato plants. Once again, we can hypothesize that the form of As and Cu released into the soil solution might have played a role in such restriction of plant uptake. Indeed, the BCS AMOchar concomitantly to the release of As and Cu also induced a leaching of DOC. This released DOC could have formed complexes with both elements, making them unavailable for plant uptake. It is also possible that those complexes were more stable in the case of BCS than BCM, explaining the discrepancies between the two materials in terms of As and Cu leaching and plant uptake. Additionally, it is possible that As and Cu competed with P for plant uptake, which restricted their entry. These results show the advantage of BCS over BCM and pristine BC in reducing the entry of metals and As into the food chain and potentially allowing the use of the biomass produced on the soil for diverse industrial and agronomical purposes.

### Stability, drawbacks, and comparison of prepared amendments

As mentioned before, MnOx are generally very efficient stabilizing amendments, but their reactivity in soil generally includes the redox reactions with the SOM and connected Mn and DOC release. However, these parameters are not often included in the remediation studies dealing with MnOx-based materials, and in many cases, the stability of the applied material and its impact on SOM thus remains unknown. Being familiar with the advantages and drawbacks of both MnOx and biochar with respect to their effects on contaminants mobility and soil environment, the AMOchars used in the current study were intentionally developed by our team over time with the aim to leverage the advantages of both materials while simultaneously mitigating their shortcomings. Firstly, a new amorphous oxide (AMO) was synthesized using glucose as the reducing agent and tested as a highly efficient immobilizing agent for Cd, Pb, Zn, and As^11,12,14^. When applied to the same soil as used in the current study, it efficiently decreased the uptake of Zn by sunflower almost by sixfold compared to the control, yet the content of Mn in shoots increased by 22-fold to the level that the Mn itself became phytotoxic^14^. For this reason, with the aim of decreasing the Mn leaching, various glucose-based AMOchars were prepared, and after the initial successful results from the sorption tests^16^, the most promising composite was tested in soil^15^. Concomitantly, sucrose-based and molasses-based AMOchars were prepared to further improve the synthesis yield and economic costs and also to reduce the Mn leaching^17^. Finally, when comparing the current results with the glucose-based AMOchar applied to the same soil as in the current paper and incubated under similar conditions^15^, both BCS and BCM showed much lower Mn release (∼1.6 fold higher than the control) compared to the glucose-based AMOchar (∼tenfold higher than control), while, at the same time, had significantly higher immobilizing efficiency towards Cd (∼threefold decrease of concentration of Cd in SPW compared to control for glucose/AMOchar, *vs*. ∼sevenfold decrease for BCS/BCM) and Zn (∼threefold decrease for glucose/AMOchar, *vs*. ∼tenfold decrease for BCS/BCM). From this perspective, among all the composite materials tested, BCS presents the best compromise between the immobilizing performance and the material’s stability.

But the use of this material needs to be caution, especially in soil with high levels of As and Cu, which will be mobilized by its addition. This material could be used when one wants to extract those elements, through the use of (hyper)accumulator plants and with the help of the BCS AMOchars which will make them more available for plants. Moreover, the Mn leaching is still a problem, as Mn could be spread to other compartments of the ecosystem (*e.g*., nearby river, underground water) and cause hazardous effects to the aquatic life and human health. Solutions need to be found to overcome this. For instance, the use of Mn-accumulating plants could help lower the amount of Mn leached from the soil. Combining AMOchars with pristine biochar could allow for the released Mn to be immobilized on the pristine biochar. Finally, the synthesis process could be further optimized to increase the stability of the MnOx on the biochar surface.

## Conclusions

The results of our mesocosm experiment demonstrated the benefits of biochar modification for the stabilization of Cd and Zn, reducing their release into the soil solution compared to pristine biochar. The impregnated biochars also showed a decrease in the transfer of those metals into the plant tissues. However, those materials had a negative influence on the release of DOC from soil, the leaching of As and Cu, and their transfer to plants. Even though all of the biochar composites failed to improve the biomass production of ryegrass, they did not present any toxicity to the plants and improved the phytostabilization of several metallic elements (*i.e*., Cd, Pb, and Zn). Furthermore, compared to our previous study, we showed that selecting a biochar with a higher surface area, and changing the sugar source in the reducing step during material synthesis significantly lowered the loss of Mn and organic carbon while improving the stabilizing effectivity. Finally, among the two materials, we demonstrated that in the real soil system, the MnOx/biochar composite BCS produced using sucrose as a reducing agent had higher benefits than the one produced using molasses, and this approach is an efficient way to improve the phytostabilization of Cd, Pb and Zn. This material could be used in the field without impairing plant establishment, therefore not retarding the stabilization of the soil metal(loid)s, even accelerating it. However, caution should be taken when applying it to soil highly contaminated with As and Cu, as their leaching could cause transfer to other compartments of the ecosystems, and ultimately cause health hazard, together with the leached Mn. In addition, BCS needs to be also tested in soils under changing redox conditions, as the decrease in Eh can further promote leaching of Mn and associated contaminants. To reduce those negative effects, additional work should be carried out, such as growing plants able to accumulate Mn, or mixing AMOchars with pristine biochar capable to bound the released Mn. Optimization of the application dose and of the synthesis process could also be done.

## Supplementary Information

Supplementary Information.

## Data Availability

Data will be made available upon request to the corresponding author.
