# Supplementary material for: AMOchar: an amorphous MnOx functionalized biochar to stabilize metal(loid)s in soil and optimize phytostabilization
Source: Sci Rep. 2025 Nov 21;15:41210. doi: 10.1038/s41598-025-25164-4 (PMC12638751; doi:10.1038/s41598-025-25164-4)
Supplement: Supplementary file 1 — Supplementary Information. [file 41598_2025_25164_MOESM1_ESM.docx]

*Supplementary Information*

**AMOchar, an improved biochar material to stabilize metal(loid)s in soil and optimize phytostabilization**

**S1 Synthesis of MnOx-biochar composites**

Synthesis of both AMOchar composites was based on the modified sol-gel procedure, which generally consisted of the reduction of KMnO_4_ by sucrose or molasses in the presence of biochar. The detailed approach is described below.

**Caution** – the reaction between the concentrated solutions of KMnO_4_ and sugars in this approach was very intensive and exothermic, so the sugar solutions had to be added to the KMnO_4_ very carefully and in small steps.

**BCS composite:**

13.3 g of KMnO_4_ was dissolved in 130 mL of deionized water (the solution needed to be heated to enable the KMnO_4_ dissolution). When the KMnO_4_ was dissolved completely, the solution was left to cool to laboratory temperature, and 6.7 g of biochar were added to the solution under constant mixing. At the same time, a solution consisting of 14.5 g of sucrose and 9.4 mL of water was prepared and slowly added by the pipette in small steps (max. 1.5 mL) to the KMnO_4_/biochar solution under constant stirring with a rod stirrer. After each step, we had to wait for a moment (approx. 5 sec) to let the mixture react before adding another sucrose. After adding all the sucrose solution, we continued the mixing until the reaction was completed (usually in the range of seconds) and the solution turned to black gel (the change from solution to partly solid gel was sudden and clearly visible). The originated gel was then filtered in the funnel with filter paper, air-dried, and ground.

**BCM composite:**

13.3 g of KMnO_4_ was dissolved in 130 mL of deionized water (the solution needs to be heated to enable the KMnO_4_ dissolution). When the KMnO_4_ was dissolved completely, the solution was left to cool to laboratory temperature, and 6.7 g of biochar were added to the solution under constant mixing. At the same time, 20.1 mL of molasses (27 g) was dissolved in 20 mL of deionized water and slowly added by the pipette in small steps (max. 2 mL) to the KMnO_4_/biochar solution under constant stirring with a rod stirrer. After each step, we had to wait for a moment (approx. 5 sec) to let the mixture react before adding another molasses. After adding all the molasses solution, we continued the mixing until the reaction was completed (usually in the range of seconds) and the solution turned to black gel (the change from solution to partly solid gel was sudden and clearly visible). The originated gel was then filtered in the funnel with filter paper, air-dried, and ground.

**Table S1** Fractionation of As, Cd, Pb and Zn in the studied soils, adapted from Michálková et al.^1^.

| **Fractionation of metals (sequential extraction by Rauret et al.^2^) (mg.kg^-1^) (*n*=3)** | | | | |
| --- | --- | --- | --- | --- |
|  | FA:  exchangeable + acid soluble | FB:  reducible | FC:  oxidizable | FD:  residual phase |
| Cd | 24.0 ± 0.3 | 8.01 ± 1.09 | 1.31 ± 0.16 | 6 |
| Pb | 281 ± 11 | 2165 ± 176 | 705 ± 93 | 388 |
| Zn | 1822 ± 50 | 816 ± 45 | 298 ± 32 | 1066 |
| **Fractionation of As (sequential extraction by Wenzel et al.^3^) (mg.kg^-1^) (*n*=3)** | | | | |
| FA:  non-specifically sorbed | FB:  specifically adsorbed | FC:  bound to amorphous and poorly crystalline hydrous oxides of Fe, Al and Mn | FD:  bound to well-crystallized hydrous oxides of Fe, Al and Mn | FE:  residual  phase |
| 0.16 ± 0.02 | 20.2 ± 0.1 | 214 ± 4 | 45.9 ± 8.4 | 16 |
|  |  |  |  |  |

**Table S2** Elemental composition of pristine biochar (BC) and AMOchars prepared using molasses (BCM) and sucrose (BCS).

|  | **Al** | **Ba** | **Ca** | **Fe** | **K** | **Mg** | **Na** | **P** | **S** | **Ti** | **Zn** |
| --- | --- | --- | --- | --- | --- | --- | --- | --- | --- | --- | --- |
|  | **(mg.kg^-1^)** | | | | | | | | | | |
| **BC** | 3647  ±  304 | 546  ±  2 | 12911 ±  97 | 5130  ±  170 | 3394  ±  57 | 2774  ±  363 | 2207  ±  15 | 561  ±  1 | 430  ±  38 | 407  ±  2 | 174  ±  1 |
| **BCM** | 772  ±  13 | 53  ±  2 | 3086  ±  150 | 880  ±  11 | 80865  ±  134 | 619  ±  17 | 3802  ±  0 | 209  ±  35 | 1757  ±  68 | 77  ±  2 | 44  ±  16 |
| **BCS** | 803  ±  1 | 57  ±  2 | 3120  ±  6 | 612  ±  21 | 82865  ±  301 | 594  ±  5 | 400  ±  9 | 149  ±  0 | 1072  ±  11 | 75  ±  0 | 21  ±  2 |

**References:**

1. Michálková, Z., Komárek, M., Vítková, M., Řečínská, M., & Ettler, V. (2016). Stability, transformations and stabilizing potential of an amorphous manganese oxide and its surface-modified form in contaminated soils. *Applied Geochemistry*, *75*, 125–136. https://doi.org/10.1016/J.APGEOCHEM.2016.10.020
2. Rauret, G., Lopez-Sanchez, J. F., Sahuquillo, A., Barahona, E., Lachica, M., Ure, A. M., Davidson, C. M., Gomez, A., Luck, D., Bacon, J., Yli-Halla, M., Muntau, H., & Quevauviller, P. (2000). Application of a modified BCR sequential extraction (three-step) procedure for the determination of extractable trace metal contents in a sewage sludge amended soil reference material (CRM 483), complemented by a three-year stability study of acetic acid and EDTA extractable metal content. *Journal of Environmental Monitoring*, *2*(3), 228–233. https://doi.org/10.1039/B001496F
3. Wenzel, W. W., Kirchbaumer, N., Prohaska, T., Stingeder, G., Lombi, E., & Adriano, D. C. (2001). Arsenic fractionation in soils using an improved sequential extraction procedure. *Analytica Chimica Acta*, *436*(2), 309–323. https://doi.org/10.1016/S0003-2670(01)00924-2
